# Spatiotemporal Expression Characterization of *KRTAP6* Family Genes and Its Effect on Wool Traits

**DOI:** 10.3390/genes15010095

**Published:** 2024-01-14

**Authors:** Hongxian Sun, Zhaohua He, Fangfang Zhao, Jiang Hu, Jiqing Wang, Xiu Liu, Zhidong Zhao, Mingna Li, Yuzhu Luo, Shaobin Li

**Affiliations:** Gansu Key Laboratory of Herbivorous Animal Biotechnology, International Wool Research Institute, College of Animal Science and Technology, Gansu Agricultural University, Lanzhou 730070, China; sunhx@st.gsau.edu.cn (H.S.); hezh@st.gsau.edu.cn (Z.H.); zhaofangfang@gsau.edu.cn (F.Z.); huj@gsau.edu.cn (J.H.); wangjq@gsau.edu.cn (J.W.); liuxiu@gsau.edu.cn (X.L.); zhaozd@gsau.edu.cn (Z.Z.); limn@gsau.edu.cn (M.L.); luoyz@gsau.edu.cn (Y.L.)

**Keywords:** hair follicle, wool traits, spatiotemporal expression, *KRTAP6s*, sheep

## Abstract

Keratin-related proteins (KAPs) are structural components of wool fibers and are thought to play a key role in regulating the physical and mechanical properties of fibers. Among all KAP genes (*KRTAPs*), *KRTAP6* gene family (*KRTAP6-1*, *KRTAP6-2*, *KRTAP6-3*, *KRTAP6-4*, and *KRTAP6-5*) is a very important member with high polymorphism and notable association with some wool traits. In this study, we used real-time fluorescence quantitative PCR (RT-qPCR) and in situ hybridization to investigate spatiotemporal expression of *KRTAP6s*. The results revealed that *KRTAP6* family genes were significantly expressed during anagen compared to other stages (*p* < 0.05). And it was found the five genes were expressed predominantly in the dermal papillae, inner and outer root sheaths, and showed a distinct spatiotemporal expression pattern. Also, it was found that *KRTAP6-1* and *KRTAP6-5* mRNA expression was negatively correlated with wool mean fiber diameter (MFD) and mean staple strength (MSS) (*p* < 0.05). In summary, the *KRTAP6* family genes share a similar spatiotemporal expression pattern. And *KRTAP6-1* and *KRTAP6-5* may regulate the MFD and MSS of Gansu Alpine fine-wool sheep wool by changing the expression.

## 1. Introduction

Wool as a natural fiber and artificial fiber have huge differences; its unique structure and chemical composition compared with other fibers have a great competitive advantage and give it a complex diversity, popular in the market [1]. It has been found that wool is a polymer of a variety of amino acids, of which there are 18, but there are only 2 for synthetic fibers and this difference in the number of amino acids also results in the chemical properties of wool fibers being superior to those of synthetic fibers [2]. At the same time, due to the unique two-layer cellular structure of wool fibers, which are the inner cells of the cortex and the outer cells of the cuticle, it is this unique structure that causes the physical properties of wool fibers (waterproofing, shrinkage, insulation, softness, etc.) in the application of other fibers to not have the same characteristics [3]. As a result, the superior chemical properties and unique physical structure of wool fibers allow wool to be used in a variety of applications. Firstly, wool is a household name in the textile industry and, nowadays, it is also being used progressively in technical applications [4]. This study found that wool technology is not only being used in traditional industries but is now being used in a number of other areas, including construction [5], air pollutant treatment [6], and heavy metals [7].

The hair follicle is the core regulatory part of wool growth and development, and the level of hair follicle development is directly related to the quality of wool [8]. Hair follicles undergo cycle after cycle of growth, which is regular, and this cycle of growth is accompanied by a number of histomorphologic changes, mainly morphological and structural changes in the hair papilla, formation of new HS, and shedding of old hairs. The hair follicle is a self-renewing organ, and this cyclic growth is divided into three periods: anagen, catagen, and telogen [9]. This cyclic activity of the hair follicle is controlled by epithelial–mesenchymal interactions between follicular keratin-forming cells and hair papilla fibroblasts [10]. Of course, this cyclic growth of hair follicles can be influenced by many factors and, among them, genetic factors are the main cause, while environment, gender, and nutrition also exist [11]. It has been found that the transition of hair follicles from anagen to resting phase can be affected and delayed by red light at 650 nm and it affects the proliferation of hair follicle cells [12]. Gender also has an effect on follicular cycle growth, which is mainly regulated by hormones, with the effect of androgens having a greater regulatory effect on hair growth [13]. At the same time, the cyclic growth of hair follicles requires the provision of nutrients to the hair papilla and the regulation of the peripheral nerves [14]. Hair follicle and wool growth and development are regulated by a variety of signaling pathways and related genes, such as the average fiber diameter as the most critical trait index of wool being regulated by genes related to early hair follicle development, and this is also highly correlated with the size of the substrate and the number of dermal papillae [15,16,17], and the traits of wool are closely related to the development of the hair follicle, so that the genetic factors are the determinants of the traits of wool.

The hair shaft is the part of the skin that is exposed and is made up of keratinocytes from the hair follicle [8]. And the hair shaft is mainly composed of two proteins, keratin and keratin-associated protein (KAPs) [18,19]. Two right-helix macromolecules are combined in a helical manner to form a left-helix molecule, several left-helix molecules are bundled together as keratin (KRT), several keratin are bundled together as microfibrils, which are externally wrapped by keratin-associated protein (KAP), and several giant fibers are wrapped in bundles by paracortical cells (which also include nuclear remnants and interstitium) and numerous pro-cortical cells. A number of keratin-associated protein-coated units were bundled together to form giant fibers, and a number of giant fibers were bundled together by paracortical cells (which also included nucleus remnants and interstitial cells), together with a number of pro-cortical cells and orthocortical cells, and sequentially wrapped by the squamous layer (inner epidermis, sub-epidermis, and outer epidermis), which formed the hair fibers [20]. It was found that keratins are α-keratins assembled in a specific way to form keratin intermediate filaments (KIFs) in the cytoplasm of hair cells while being encapsulated in a filamentous matrix composed of KAPs [21]. KAPs have a relatively small molecular weight and are mostly encoded by a large number of multigene families (*KRTAPs*), while the amino acid content contained in KAPs has been categorized into three groups [22,23]. Different breeds of wool produced by sheep have large differences in the quality of wool; physical indicators of sheep wool quality traits are mainly fineness, followed by length, strength and elongation, curvature, etc., and production practice is often based on the main trait indicators to measure the quality and economic value of sheep wool. A large number of *KRTAPs* genes have been identified in humans and other mammals during the last few decades of research, while much of the research on these genes has focused on the study of human hair and sheep wool traits [24]. It was found that *KRTAPs* have an important role in the maintenance of the physical and mechanical properties of wool fibers [25]. *KRTAP6* is a member of the HGT-KAP family, and three family members have been identified in humans, while *KRTAP6-1* and *KRTAP6-2* were identified in early studies in sheep and three more family members, *KRTAP6-3*, *KRTAP6-4*, and *KRTAP6-5*, have been identified since then [26]. It was found that the variation in *KRTAP6-1* and *KRTAP6-3* had an effect on the mean fiber diameter (MFD) of sheep [27,28].

Given that *KRTAP6* family genes play an important regulatory role in hair follicle development and hair growth, analyzing their spatiotemporal expression characteristics and their relationship with wool traits is of great significance to improve wool quality. Therefore, in this study, the mRNA expression and expression location of *KRTAP6* family genes in Gansu Alpine fine-wool sheep were investigated by in situ hybridization and real-time fluorescence quantitative PCR (RT-qPCR). The *KRTAP6* family genes were analyzed, which laid the foundation for the regulatory mechanism of wool growth and provided the basis for the study of sheep improvement.

## 2. Materials and Methods

### 2.1. Animal Samples

In the same Gansu Alpine fine-wool sheep population in Tianzhu Tibetan Autonomous County, we randomly selected six newborn lambs for continuous tracking and collected lamb skins at six periods: 1 day, 30 day, 60 day, 90 day, 180 day, and 270 day. In addition, we randomly selected 75 Gansu Alpine fine-wool sheep (female) and collected skin and wool samples from them when they were one year old. All sheep come from the same population. Skin tissues and wool were taken from the posterior edge of the scapula within an area of 5 cm^2^ of skin. The sample was collected in June, during which the hair follicles were in the growth phase. One part of the skin sample is washed with distilled water, stored in a tank of liquid nitrogen, and brought back to the laboratory at −80 °C. In the other part, the skin was immediately rinsed with Phosphate Buffered Solution (PBS) and then placed in paraformaldehyde fixative (PFA) for cryopreservation. Wool traits were measured by the New Zealand Pastoral Measurements LTD. (Ahuriri, Napier, New Zealand). Mean fiber diameter (MFD): the general use of fiber diameter size or quality of the number of branches to indicate the thickness of wool, with the size of the fiber diameter measured by the cross-section of wool fibers; comfort factor (CF): refers to the proportion of wool in the diameter of the fiber that is less than 30 µm. Mean stable length (MSL): wool fibers have a natural curl and, in the natural curl, the distance of the straight line between the two ends is called the natural length; mean stable strength (MSS): the wool fibers used in the force are pulled off, that is, the wool fibers resistant to breakage; mean fiber curvature (MFC): the natural periodic curling of wool along its length, where the degree of curling is expressed in terms of curvature per millimeter, called curvature (°/mm).

### 2.2. In Situ Hybridization Analysis

The tissue was removed, washed, and immediately placed in an in situ hybridization fixative at 4 °C for 12 h or more. The slices were prepared with a paraffin slicer and baked in an oven at 62 °C for 2 h. The slices were put into Eco-Dewax Clear I (Wuhan Xavier Biotechnology Co., Ltd., Wuhan, China) for 15 min, Eco-Dewax Clear II (Wuhan Xavier Biotechnology Co., Ltd., Wuhan, China) for 15 min, anhydrous ethanol I for 5 min, anhydrous ethanol II for 5 min, 85% alcohol for 5 min, 75% alcohol for 5 min, and then immersed in DEPC water. Sections were repaired in repair solution and then cooled naturally. Proteinase K (20 µg/mL) was added dropwise at 37 °C for digestion. Prehybridization solution was added dropwise and incubated at 37 °C for 1 h. The prehybridization solution was poured off, the hybridization solution containing the probe was added dropwise, and sections were hybridized overnight in a thermostat. The hybridization solution was washed off and they were incubated for 10 min at 37 °C for 2 × SSC, 2 × 5 min at 37 °C for 1 × SSC, and 10 min at room temperature for 0.5 × SSC. The sections were gently shaken dry, drops of prewarmed corresponding branching probe hybridization solution were added (Table 1), and they were placed horizontally in a wet box at 40 °C for 45 min. The water in the hybridization solution was removed and the sections were rinsed sequentially with 2 × SSC, 1 × SSC, 0.5 × SSC, and 0.1 × SSC at 40 °C for 5 min preheating. Hybridization solution containing the signaling probe (Table 1) was added dropwise at a dilution ratio of 1:400. Sections were then incubated at 42 °C for 3 h. The following SSC rinses were then performed sequentially: 2 × SSC at 37 °C for 10 min; 1 × SSC at 37 °C for 2 × 5 min; and 0.5 × SSC at 37 °C for 10 min. Sections were incubated with DAPI stain for 8 min, rinsed, and sealed with a drop of fluorescence quenching sealer. The sections were observed under a fluorescence microscope and images were taken.

### 2.3. RT-qPCR Analysis

Trizol method (Thermo Fisher Scientific Co., Ltd., Shanghai, China) was used for the extraction of total RNA from the collected skin samples. The purity and concentration of the extracted RNA were then determined using ultraviolet spectrophotometer and subsequently stored at −80 °C. RNA reverse transcription was performed using the Prime ScriptTM RT kit (Nanjing Novizan Biotechnology Co., Ltd., Nanjing, China) according to the manufacturer’s instructions, and the cDNA was then stored at −20 °C. β-actin was selected as the internal reference gene. All primers and PCR information are shown in Table 2. RT-qPCR was carried out using Biosystems QuantStudio^®^ 6 Flex (Thermo Lifetech, Waltham, MA, USA) and SYBR Green Pro Taq HS qPCR Kit (Accurate Biology, Changsha, China).

### 2.4. Measurements and Statistical Analysis

In situ hybridization was scanned and imaged using PANNORAMIC (3DHISTECH Ltd., Budapest, Hungary) panoramic slice scanner. The cumulative optical density (IOD) values of six positive fields in each slice were measured by Image-Pro 6.0 software (Media Cybernetics Inc., Rockville, MD, USA). The corresponding positive pixel area (Area) and AOD = IOD/Area were calculated. Positive scoring was performed on five selected sites within three fields of view per section. For RT-qPCR, the raw data were first processed using excel 2019 software and then calculated after the commonly used −2^ΔΔCT^ [29] method. All results of this study were analyzed using one-way ANOVA method by SPSS 22.0 (SPSS Inc., Chicago, IL, USA) statistical software, multiple comparisons using the Duncan method, and the results are expressed as mean ± standard deviation (S.D.), and different lowercase letters are indicated as significant (*p* < 0.05). Correlations were analyzed using Pearson’s correlation analysis, and correlations were considered significant at *p* < 0.05.

## 3. Results

### 3.1. Expression of KRTAP6 Family Genes in Different Stages of Follicle Cycle Development of Skin

**Figure 1:**
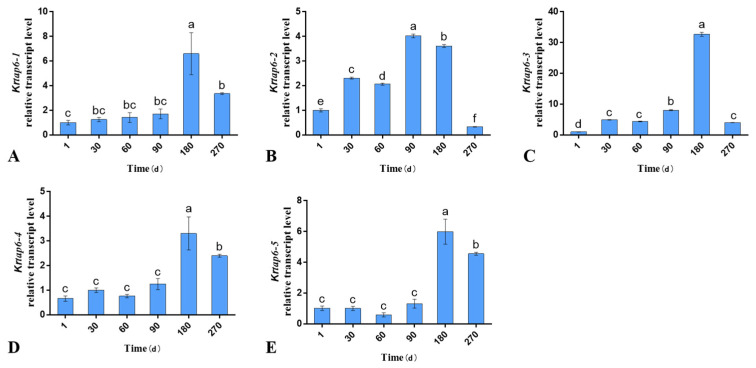
Phase mRNA levels of *KRTAP6* family genes in different stages of hair follicle development. All related genes were normalized using *β-actin*. (**A**–**E**): relative expression of *KRTAP6* family genes in the hair follicle cycle. The data were expressed as mean ± S.D. Different letters indicate significant differences by age (*p* < 0.05).

The results of fluorescence quantification showed that the KRTAP6 family genes were expressed in the skin at different stages of hair follicle cycle development. The expression of mRNA at different stages varied and showed a strong spatiotemporal expression profile. The results showed that the mRNA levels of *KRTAP6-1*, *KRTAP6-3*, *KRTAP6-4*, and *KRTAP6-5* in 180-day-old skin were the highest in the six periods (*p* < 0.05; Figure 1); *KRTAP6-2* mRNA level in 90-day-old skin was the lowest among the six periods (*p* < 0.05; Figure 1).

### 3.2. AOD Values of KRTAP6 Family at 180 Days of Hair Follicle in the Skin

**Figure 2:**
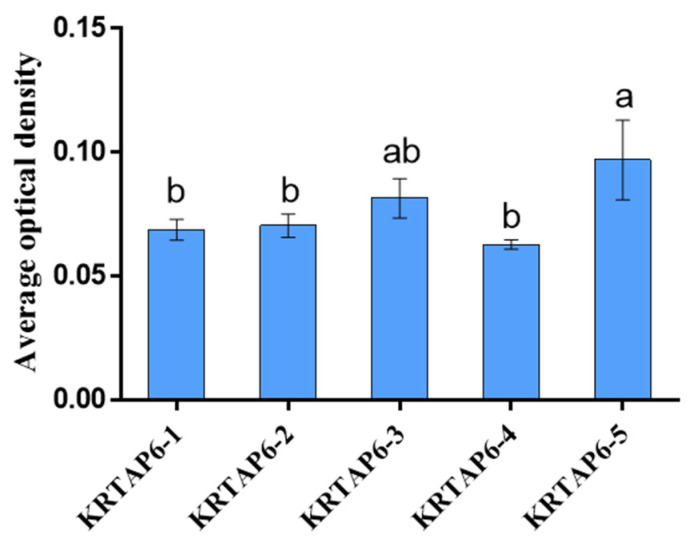
AOD values of KRTAP6 family at 180 days of hair follicle in the skin. The data are presented as mean ± S.D. Different letters indicate significant differences between ages (*p* < 0.05).

The expression of *KRTAP6* family mRNAs was calculated by in situ hybridization technique using AOD statistics (AOD = IOD/Area) in the skin of 180 days (catagen) of hair follicle cycle development (Figure 2). The results showed that the mRNA expression of *KRTAP6-5* was significantly higher than that of other family members in Gansu Alpine fine-wool sheep skin at 180 days (*p* < 0.05).

### 3.3. Distribution and Localization of KRTAP6 Family in 180 Days of Hair Follicle Cycle Development in the Skin

**Figure 3:**
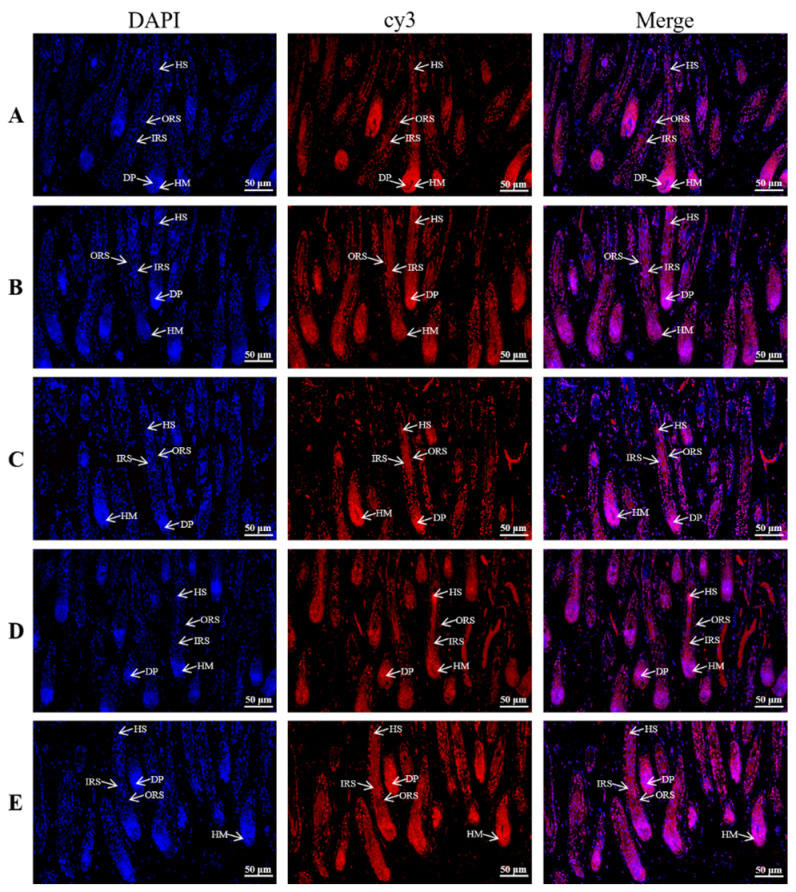
In situ hybridization staining (20×) of the KRTAP6 family at 180 days of hair follicle cycle development in the skin. (**A**) *KRATP6-1*; (**B**) *KRATP6-2*; (**C**) *KRATP6-3*; (**D**) *KRATP6-4*; (**E**) *KRATP6-5*. The blue tissue in the figure shows DAPI-labeled nuclear fluorescence staining and the red tissue shows fluorescence staining of cy3. ORS: outer root sheath; IRS: inner root sheath; DP: dermal papilla; HM: hair matrix; HS: hair shaft.

The intensity of positivity of the *KRTAP6* family genes at different sites of skin follicle cycle development for 180 days was analyzed using in situ hybridization (Figure 3). Positive scoring results showed that *KRTAP6-1* had medium positive expression in the corneum, strong positive expression in the inner and outer root sheaths, weak positive expression in the hair medulla, and high-density strong positive expression in the dermal papilla (Table 3). The positive scores showed that *KRTAP6-2*, *KRTAP6-3*, *KRTAP6-4*, and *KRTAP6-5* were not positively expressed in the corneum and hair medulla but had strong positive expression in the inner root sheath and high-density strong positive expression in the outer root sheath and dermal papilla (Table 3). According to the positive results, the *KRTAP6* family genes play an important role in hair follicle development and maintenance, and this is regulated mainly through the dermal papilla and the inner and outer root sheaths.

### 3.4. Association Analysis of mRNA Expression Levels of KRTAP6 Family with Wool Traits

**Figure 4:**
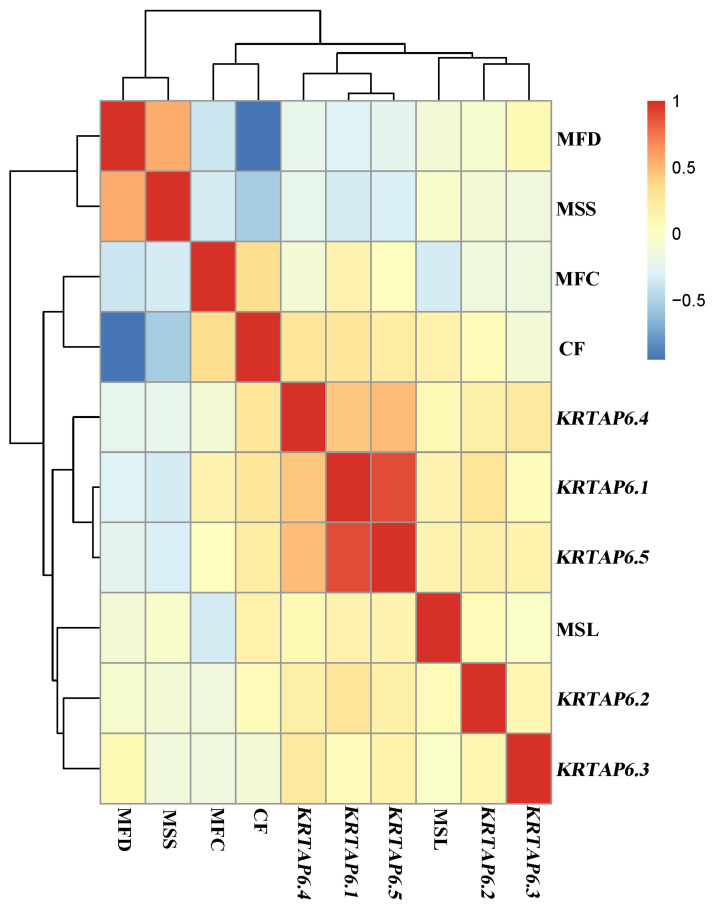
Association analysis of mRNA expression levels of *KRTAP6* family with wool traits. MFD: mean fiber diameter; MFC: mean fiber curve; MSS: mean staple strength; MSL: mean staple length; CF: comfort factor. The graph shows positive correlation in red and negative correlation in blue (*p* < 0.05).

Association analysis of *KRTAP6* family gene expression with wool traits in Gansu Alpine fine-wool sheep was performed (Figure 4). The results showed a positive correlation between *KRTAP6-1* and *KRTAP6-5* mRNA expression. And *KRTAP6-1* and *KRTAP6-5* mRNA expression was found to be negatively correlated with MFD and MSS (*p* < 0.05).

## 4. Discussion

As one of the important raw materials for the textile industry, wool products are favored by the majority of consumers because of their softness, good elasticity, moisture absorption, good thermal insulation, comfortable wearing, good abrasion resistance, and beautiful appearance [3]. The development of the wool market has stimulated the demand for different grades of wool, and the quality of wool directly affects the development of sheep farming and the wool textile industry. Therefore, improving wool production and quality can not only meet people’s increasing demand and become an effective means to increase farmers’ and herdsmen’s income but also improve the competitiveness of wool in the economic market.

In this study, we found that the expression trends of *KRTAP6-1*, *KRTAP6-3*, *KRTAP6-4*, and *KRTAP6-5* were consistent in the skin tissues of Gansu Alpine fine-wool sheep at different stages, all showing the highest mRNA expression at 180 days of age (anagen) by RT-qPCR. Hair follicle development is divided into three periods, the anagen phase from May to November, the catagen from December to January, and the telogen roughly from February to April [30]. The anagen is the longest of the three periods of hair follicle development, and this suggests that the *KRTAP6* family of genes has a very important role in maintaining wool growth. Meanwhile, in a study on Angora rabbits, it was found that, for *KRTP11-1*, localization was mainly in mitochondria, and its mRNA expression in the skin was the highest and it also showed a strong spatiotemporal expression profile, increasing and then decreasing over time, and was highly expressed in the follicle during the catagen and telogen of the hair follicle [31]. It was found that gene expression of *KRTAP9-2* and *KRTAP11-1* was mainly regulated by *Hoxc13*. Another study showed that the expression pattern of keratin genes during the hair follicle developmental cycle is similar to that of *Hoxc13* [32], while the promoter activity of some keratin proteins is affected by transcription factors, resulting in up- or down-regulation [33].

In this study, the *KRTAP6* family gene was found to be significantly expressed in Gansu Alpine fine-wool sheep at 180 d (anagen) after birth by RT-qPCR. This suggests that the *KRTAP6* family of genes plays an important role in the development of the hair follicle. Therefore, we further investigated the *KRTAP6* family genes using in situ hybridization and found that the expression of *KRTAP6-5* mRNA in Gansu Alpine fine-wool sheep skin was significantly higher than that of other family members at 180 d. This also suggests that *KRTAP6-5* may be an important member in the *KRTAP6* family. Meanwhile, in a study on Angora rabbits, it was found that *KRTP11-1* mRNA showed a strong spatiotemporal expression profile in the skin, and it was highly expressed in the degenerative phase of the hair follicle [31]. In another study in sheep, *KRTAP3-3* and *KRTAP11-1* mRNA were found to be expressed in a tissue-specific manner, especially in the hair follicle [34]. Our localization studies of five genes of the *KRTAP6* family revealed regulation mainly in the dermal papilla of the hair follicle and the inner and outer root sheath. Meanwhile, in another experiment on *KRTAP6* in situ hybridization, it was found that *KRTAP6* showed a clear hybridization signal at nearly 200 μm above the cortical layer of the proliferative zone of hair follicle cells, but no hybridization signal was detected in the epidermis and other types of hair follicle cells [35]. In studies on humans, *KRTAP6* was found to be expressed in all cortical layers, while, in sheep, it was found to be expressed only in the orthocortical layer [36]. Moreover, *KRTAP6* was found to have a highly different expression pattern in sheep and humans, with high expression in sheep and low expression in humans [19,37]. This finding also indicates that *KRTAP6* plays an important role in hair follicle development and hair maintenance. Localization of the distribution of *KRTAP1* and *KRTAP26* in studies of human hair revealed that *KRTAP1* is present in cortical cells and *KRTAP26* in the outer and inner epidermis [38]. Sheep *KRTAP11-1* was found to be highly expressed in wool cortex by in situ hybridization [34], whereas, in studies on Angora rabbits, *KRTAP11-1* was found to be a hydrophilic protein mainly located in mitochondria [31]. In contrast, *KRTAP24-1* characterized in humans was found to be specifically expressed in the hair cuticle [39]. It was also found that *KRTAP2* was significantly expressed in the keratinized areas of the human hair shaft cortex [18]. In merino hair follicles, activation of the hair *KRTAPs* gene has been reported to occur in the cortical region [40].

In addition, to further investigate the relationship between *KRTAP6* expression and wool traits, this study analyzed the association between mRNA expression of *KRTAP6* family genes and wool traits and found that *KRTAP6-1* and *KRTAP6-5* were significantly negatively correlated with MFD and MSS. Mean fiber diameter, statistical data describing the fineness of wool, is also an important factor in determining the yarn count, i.e., the finer the wool fiber content, the less tingling the fabric made will feel when worn and, vice versa, the greater the tingling sensation on the skin [41]. Mean staple strength is an important indicator of the mechanical properties of wool but also determines the strength of woolen products and the production of the most essential factors of the use of woolen products and can directly affect the woolen products in terms of the sturdiness of the woolen products; if the strength of wool is not enough, it generally cannot be used as combed wool [42]. In the study of *KRTAP6* family gene variation, *KRTAP6-1* was found to be polymorphic and significantly associated with MFD [27], and *KRTAP6-3* was found to be significantly associated with MFD as well [28], which is also consistent with the results of the present study. It was found that *KRTAP6* family members are localized to chromosome 1 and quantitative trait loci (QTL) associated with MFD were identified [43]. In studies on goats, the *KRTAP6-2* polymorphism was found to have an effect on MFD [44]. An association with MSL and MFC was found in a study of the *KRTAP6-1* polymorphism in beach goats [45]. Interestingly, the present study found that *KRTAP6-1* and *KRTAP6-5* were also associated (negatively) with MSS, which was not reported in previous studies. It was found that there were significant differences between MFD and MSS in different parts of different breeds of sheep, while wool MFD and MSS were found to be positively correlated [42]. At the same time, the team’s study of the wool parameters of the sheep selected in the article revealed a range of MFD (19.70–27.54 μm) and MSS (9.47–13.75 cm), and this suggests an important role for future breeding.

In conclusion, the expression of *KRTAPs* genes is tightly regulated, and the pattern of regulation is extremely complex, with many *KRTAPs* genes having different activation and expression regions, which work together to maintain hair follicle development. This study also confirms that *KRTAP6* family genes have temporal and spatial expression patterns, indicating the importance of studying the developmental mechanisms of *KRTAP6* family genes. It was also found that *KRTAP6-1* and *KRTAP6-5* mRNA expression was significantly positively correlated and negatively correlated with wool MFD and MSS, which also indicated that the *KRTAP6* family of genes had an important influence on wool traits and could be further studied for the production of practical improved varieties to enhance economic benefits.

## 5. Conclusions

In conclusion, this study showed that (1) *KRTAP6* family genes showed high expression in the hair follicle anagen and played important roles in the hair follicle cycle in Gansu Alpine fine-wool sheep; (2) *KRTAP6* family genes are mainly expressed in outer root sheaths, inner root sheaths, and dermal papillae of Gansu Alpine fine-wool sheep to realize the regulation of wool growth and maintenance of structure; (3) *KRTAP6-1* and *KRTAP6-5* had a significant negative regulatory relationship with wool MFD versus MSS.

## Figures and Tables

**Table 1:** KRTAP6 family genes’ in situ hybridization probe information.

Probe Name	Probe Sequence (5′–3′)	Probe Concentration (nM)	Hybridization Temperature (°C)
*KRTAP6-1*	GAGGGCACGAAGCAAGTCTTTAGTAGG	500	40
*KRTAP6-2*	GTTCAGTATACGTGGTGTTCAGAACTGG	500	40
*KRTAP6-3*	ATGATGCAGCCTAACCATCACCCAGGG	500	40
*KRTAP6-4*	TGTAGTGTTGTGCATCATCTCAGGGT	500	40
*KRTAP6-5*	GTTAAAAGGTAGGCTAGGAATAGTCCA	500	40

**Table 2:** Primer sequences and annealing temperatures used for RT-qPCR.

Gene	GenBank Accession No.	Primer Sequence (5′–3′)	Product Length (bp)	Annealing Temperature (◦C)
*KRTAP6-1*	NM_001193399.1	F: TGCATGGAAGTCAAAAGAGAGTT	154	60
		R: CCTTCCGGTGCCCTCTACAT		
*KRTAP6-2*	KT725832.1	F: CCTCGGCTGTGGAAGCTAT	174	60
		R: TAGCCACAGCCATAGAGAGG		
*KRTAP6-3*	MF061690.1	F: CCTCGGCTGTGGAAGCTAT	128	60
		R: CAGAGCCATAGCCGCATC		
*KRTAP6-4*	KT725840.1	F: TCTCCTCCATCCAAGAACAACC	155	60
		R: TCCATAGCCATAGCTTCCACAG		
*KRTAP6-5*	KT725846.1	F: CCTTTTTCCCTGGGTGATGC	199	60
		R: GACAAAGACAGCATGGAAGGC		
*β–actin*	NM_001009784	F: AGCCTTCCTTCCTGGGCATGGA	113	60
		R: GGACAGCACCGTGTTGGCGTAGA		

**Table 3:** Positive intensity of KRTAP6 family in various parts of skin tissue at 180 days.

Days	Corneum	Inner Root Sheath	Outer Root Sheath	Hair Medulla	Dermal Papilla
*KRTAP6-1*	++	+++	+++	+	++++
*KRTAP6-2*	−	+++	++++	−	++++
*KRTAP6-3*	−	+++	++++	−	++++
*KRTAP6-4*	−	+++	++++	−	++++
*KRTAP6-5*	−	+++	++++	−	++++

Note: −, no positive expression; +, weak positive expression; ++, medium positive expression; +++, strong positive expression; ++++, high-density strong positive expression.

## Data Availability

The authors affirm that all data necessary for confirming the conclusions of the article are present within the article, figures, and tables.
